# Suspected chlamydial foetal loss highlights the need for standardised on‐farm protocols

**DOI:** 10.1111/avj.13206

**Published:** 2022-09-07

**Authors:** SI Anstey, C Jenkins, M Jelocnik

**Affiliations:** ^1^ Centre for Bioinnovation University of the Sunshine Coast Sippy Downs Queensland Australia; ^2^ NSW Department of Primary Industries, Animal and Plant Health laboratories Elizabeth Macarthur Agricultural Institute Menangle New South Wales Australia

**Keywords:** *Chlamydia psittaci*, foetal losspoint of care testingthoroughbred studzoonosis

## Abstract

*Chlamydia psittaci* is a recognised cause of late‐term equine foetal loss and poses a zoonotic risk in Australia. However, a management strategy is lacking to protect at‐risk humans handling infected aborted material and pregnant mares. This study proposes a protocol for approaching *C. psittaci* foetal loss after investigating four foetal losses that occurred on a horse stud in the Hunter Valley, Australia in 2021. Swabs from the foetal loss cases (*n* = 4), close contact mares (*n* = 59), and foals of the close contact mares (*n* = 33) were collected and tested for *C. psittaci* using both isothermal points of care and quantitative polymerase chain reaction (qPCR) laboratory‐based testing. Genotyping was performed utilising *C. psittaci* multilocus sequence typing and *omp*A sequencing from *C. psittaci* positive pooled foetal and placental (*n* = 3) DNA. Foetal and placental samples from the four foetal loss cases were all positive for *C. psittaci* with 100% agreement between the isothermal swab testing on the farm and qPCR DNA testing at an external laboratory. Genotyping revealed the clonal and identical sequence type 24 (ST24) *C. psittaci* strains in all samples. *C. psittaci* was not detected in close contact with mares or their foals. There was no statistically significant difference in foal survival between the close contact mare groups that did and did not receive antimicrobial intervention (P > 0.05). The proposed protocol is intended to raise awareness and begin a discussion for guidelines around handling of chlamydial foetal loss cases in late pregnant mares which pose a zoonotic threat to farm workers and veterinarians.

**List of abbreviations**
POC: point of care
qPCR: quantitative polymerase chain reaction
ST: sequence type

In addition to well‐known causes of reproductive loss such as equine herpesvirus‐1 and 4 (EHV‐1/4), *Chlamydia psittaci* (*C. psittaci*) is now also a recognised cause of late‐term equine foetal loss and poses a zoonotic risk in Australia.^1^, ^2^, ^3^, ^4^ *C. psittaci* infections in horses are postulated spill‐over events from avian reservoir hosts to late pregnant mares.^3^, ^4^ Whilst advances in understanding this pathogen in the equine have been made since its diagnosis in 2014,^1^, ^2^ a management strategy is needed to protect at‐risk humans handling infected aborted material and pregnant mares. This study reports on four *C. psittaci* foetal losses that occurred on a single horse stud in the Hunter Valley, New South Wales (NSW), Australia, in the 2021 foaling season (Figure 1). Based on this study, we propose an on‐farm management strategy for *C. psittaci* foetal loss.

**Figure 1:**
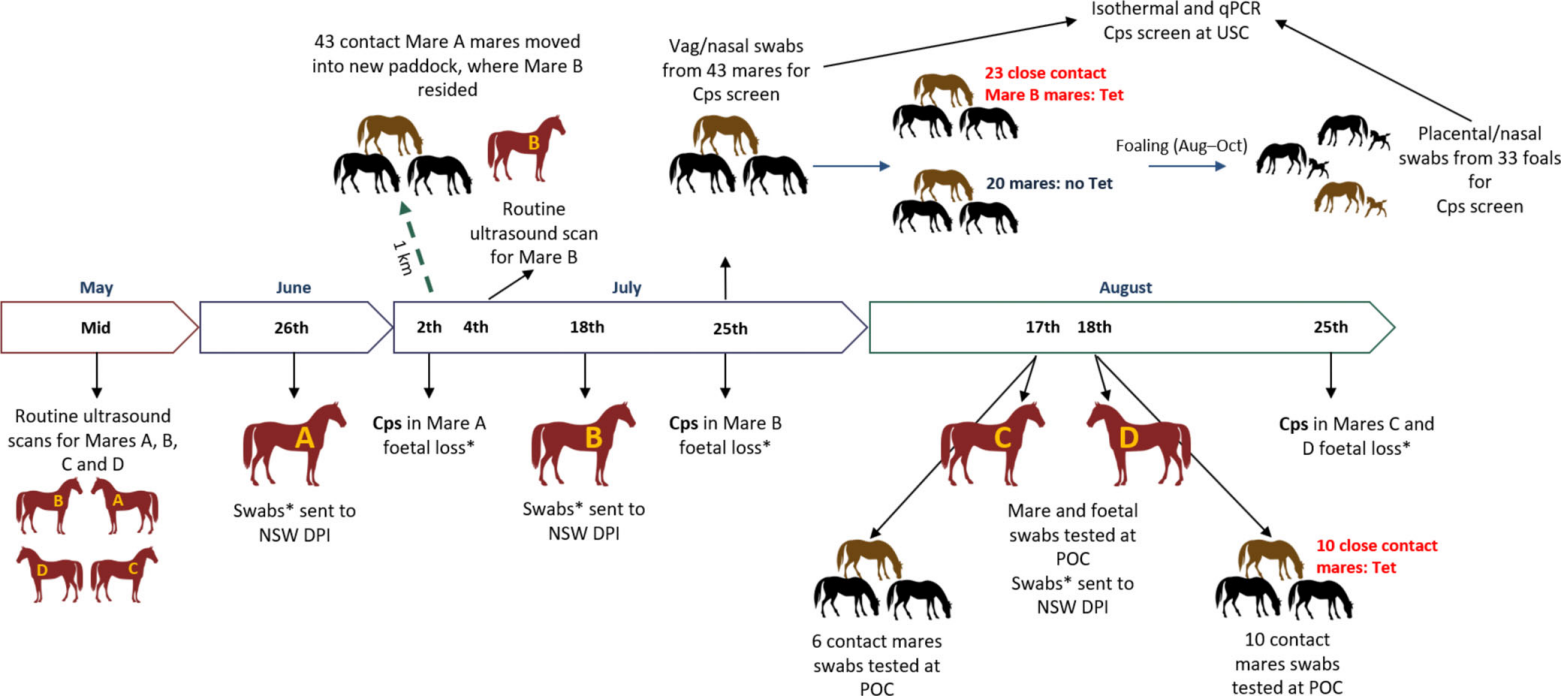
Timeline of the four foetal loss cases on a stud. Dates of the foetal loss for each mare, movement of close contact mares, swab collections, testing methods and results, and tetracycline (Tet) administration are outlined on the timeline. *Placental and foetal swabs only tested in NSW Animal and Plant Health laboratories, where *C. psittaci* (Cps) diagnosis was made.

The farm examined in this study had a history of chlamydia‐associated foetal in previous years where at least two mares had previously suffered a chlamydia‐associated foetal loss; however, the four aborting mares in this study (Mare A, Mare B, Mare C and Mare D) had no previous history of foetal loss. These mares were resident on the farm, multiparous with an average gestation length of between 335 and 365 days, current for EHV‐1 vaccinations, were not prescribed any medical therapies during the current pregnancies and recorded uneventful routine ultrasound scans in May 2021. No aborting mares showed any signs of impending foetal loss (no cervical discharge and premature mammary development). Supplementary feeding was provided as a mix of pellets, lucerne, oaten chaff and oats given once per day, and then increased to twice per day as foaling neared. All paddocks had lush green grass, and two large flocks of galahs (*Eolophus roseicapilla*) and sulphur‐crested cockatoos (*Cacatua galerita*) were anecdotally observed in Mare A's paddock during late pregnancy. Samples from this study were collected by the farm veterinary team for diagnostic purposes. Ethical approval was granted by the University of the Sunshine Coast (USC) Animal Ethics Committee (Approval numbers ANE19149; ANE1719 and ANE1939).

As outlined in Figure 1, Mare A aborted on June 26th, 2021, at 297 days gestation and swabs were taken of the aborted foetus and placenta and sent to the NSW Animal and Plant Health Laboratories at the Elizabeth Macarthur Agricultural Institute (EMAI) for *C. psittaci* quantitative polymerase chain reaction (qPCR) testing. Upon confirmation of a diagnosis of *C. psittaci* on 2nd July 2021, 43 at‐risk contact mares were moved to a new paddock 1 km away, where Mare B resided. Mare B was aborted on 18th July 2021, at 296 days of gestation and 22 days after the first case of chlamydial foetal loss. In addition, Mare B was scanned 2 weeks before her foetal loss event and no abnormalities were detected (Figure 1). Swabs were taken from the aborted foetus and placenta and sent to EMAI for *C. psittaci* qPCR testing and a second diagnosis of *C. psittaci* foal loss was recorded on 25th July. This resulted in precautionary rectal and abdominal scanning of the 43 at‐risk mares and no abnormalities were detected. Twenty‐three mares, considered close contacts of Mare B, were treated with tetracycline twice a day for 10 days at 7 mg/kg (Coopers, NSW, Australia). The remaining 20 mares were not given tetracycline. At the same time, nasal and vaginal swabs for *C. psittaci* detection were collected from all 43 contact mares.

At parturition, 33 of the 43 foals were sampled and the remaining foals (*n* = 10) were not sampled due to logistical challenges. Of the foals that were sampled, paired nasal and placental swabs were taken from 27 foals, individual nasal swabs were taken from four foals and two foals only had placental swabs. At the USC laboratory, pooled nasal and vaginal swabs (*n* = 43) from 43 mares and pooled and/or 33 individual foal swabs were processed and tested with the rapid *C. psittaci* isothermal assay^5^ (Table 1), followed by DNA extraction and *C. psittaci*‐specific (targeting the 263 bp of the conserved Cps_0RF_0607) and equine (targeting 168 bp of the equine *Cyt*B gene) DNA qPCR testing on extracted DNA from the same samples^1^, ^5^ (Table 1).

**Table 1:** *Chlamydia psittaci* detection utilising isothermal and qPCR assays of foetal loss cases, close contact mares and foals from a stud farm in the Hunter Valley, Australia and during the 2021 foaling season

ID	Swab site sample	External laboratory^a^		Point of care laboratory^c^
		*C. psittaci* LAMP	*C. psittaci* qPCR	*C. psittaci* LAMP
Foal A	Pooled foetal and placental tissues^e^		POS^b^ ^,^ ^c^	
Placenta A				
Foal B	Pooled foetal and placental tissues^e^		POS^b^ ^,^ ^c^	
Placenta B				
Mare A & Mare B close contacts mares (*n* = 43)	Pooled nose and vagina	NEG^c^	NEG^c^	
Mare A & Mare B close contact foals (*n* = 27)	Pooled nose and placenta	NEG^c^	NEG^c^	
Mare A & Mare B close contact foals (*n* = 4)	Nose	NEG^c^	NEG^c^	
Mare A & Mare B close contact foals (*n* = 2)	Placenta	NEG^c^	NEG^c^	
Mare C	Pooled nose and vagina^d^			NEG
Foal C	Pooled nose, vagina and third eyelid		POS^b^ ^,^ ^c^	POS
Placenta C	Placenta		POS^b^ ^,^ ^c^	POS
Mare C close contacts mares (*n* = 6)	Pooled nose and vagina			NEG
Mare D	Pooled nose and vagina			POS
Foal D	Pooled placenta, rectum and third eyelid		POS^b^	POS
Placenta D				
Mare D close contacts mares (*n* = 10)	Pooled nose and vagina			NEG

^a^ External laboratory, where.

^b^ NSW Animal and Plant Health laboratories and/or.

^c^ USC research laboratory.

^d^ Point of care laboratory is the stud farm's laboratory.

^e^ Pooled foetal tissues: liver, lung, spleen and thymus. Shading indicates that same samples were tested with both isothermal and qPCR *C. psittaci* assay. Mare A and Mare B were not sampled post foal loss. Ten of the 43 foals and placentas were not sampled for contact mares of Mares A and B. No foals and placentas were sampled for Mares C and D close contact mare group. Detailed results of all isothermal and qPCR testing can be found in File S1.

On 17th and 18th August 2021, Mare C and Mare D aborted at 315 and 345 days of gestation, respectively (Figure 1). These mares were residing in separate paddocks, more than 5 km away from Mare A and Mare B. Swabs were collected from the foetuses of Mare C and Mare D (Foal C and Foal D, respectively), and included nasal (*n* = 2), third eyelid (*n* = 2), vaginal (*n* = 1, Foal C), rectal (*n* = 1, Foal D) and placenta (*n* = 2, Foal C and D) swabs. At that time, the farm had point of care (POC) isothermal diagnostic capabilities and tested these rapidly processed pooled foetal swabs and an individual placental swab from Foal C (*n* = 2) and pooled foetal and placental swabs (*n* = 1) from Foal D with the rapid *C. psittaci* isothermal assay, as previously described.^5^ POC testing results were that swabs from both aborted foetuses (Foal C and Foal D) were positive for *C. psittaci*. These rapidly processed swab suspensions were also sent to EMAI for *C. psittaci* qPCR testing and a third and fourth diagnosis of *C. psittaci* foal loss was recorded, agreeing with the POC results.

Swabs of Mare C and Mare D's nose (*n* = 2), and vagina (*n* = 2) were also collected. In addition, six and ten close contacts of Mare C and Mare D, respectively, were immediately swabbed at the nasal (*n* = 16) and vaginal (*n* = 16) sites. These pooled nasal and vaginal swabs (*n* = 18) from Mares C, D and their 16 close contacts mares were also rapidly processed and tested at the POC with the rapid *C. psittaci* isothermal assay, as previously described,^5^ with only Mare D also recorded a positive result on the pooled nasal and vaginal sample (Table 1). The close contact group of Mare D (*n* = 10) were given tetracycline as previously described (Appendix S1a). Statistical comparison of the pregnancy outcome (foal live/dead) and treatment regimen of the close contact mare group, where a total of 33 mares received tetracycline and 26 were not treated, was performed using a chi‐squared 2 × 2 contingency table.

DNA extracted at EMAI from pooled foetal and placental (*n* = 3) samples from the foetuses of Mare A, Mare B and Mare C (Foal A, Foal B and Foal C) was shared for genotyping performed in this study. To determine the genotype of *C. psittaci* on a farm, multilocus sequence typing and *omp*A sequencing were performed as described in Anstey et al.^6^ (Appendix S1b) (Figure 2).

**Figure 2:**
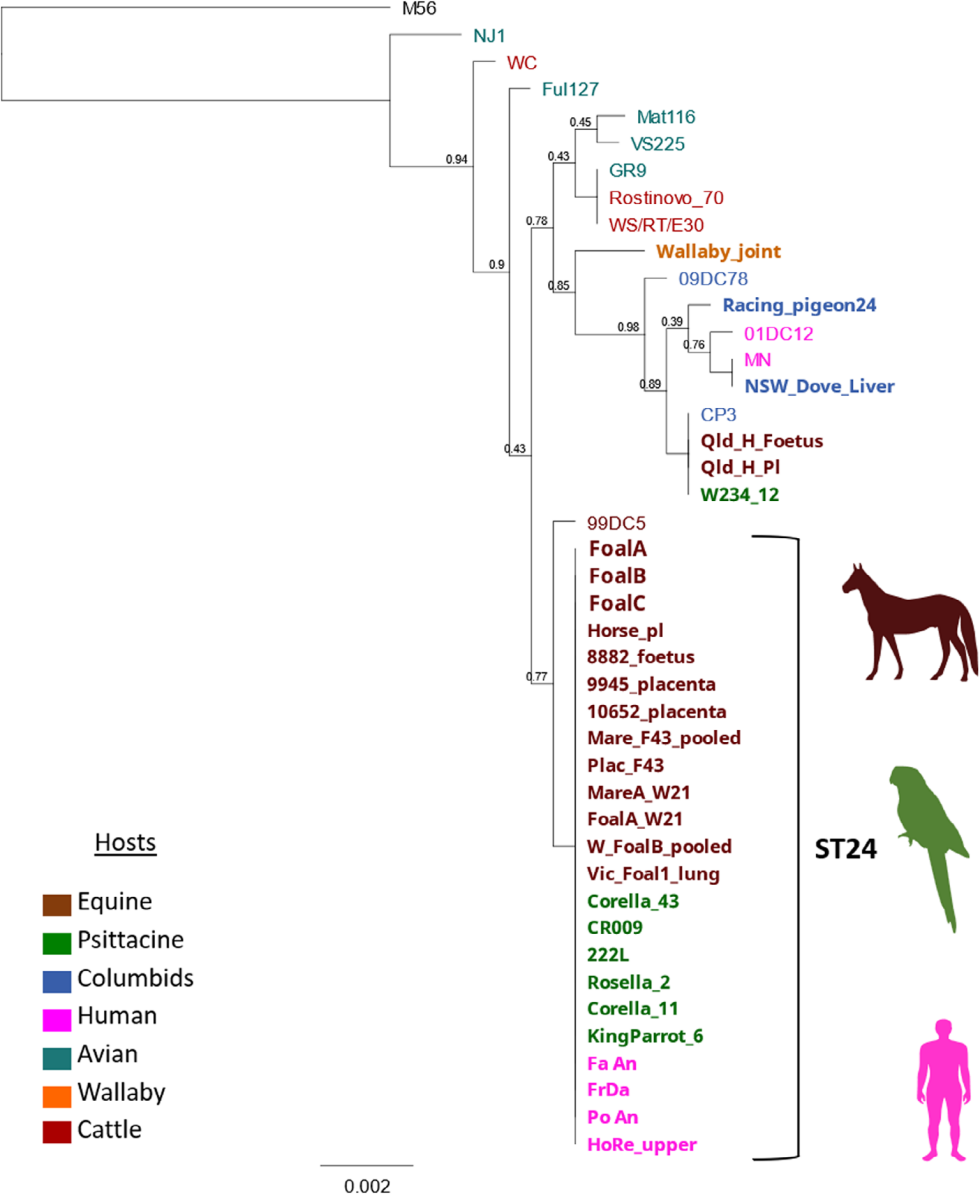
Phylogenetic analysis of 3,098 bp concatenated *C. psittaci* MLST alignments, including the three *C. psittaci* strains detected on the farm from this study in 2021. The hosts are denoted in different colours coded in the legend, and strains from this study (Foal A, Foal B and Foal C) are outlined in bold in larger font. Other Australian *C. psittaci* strains are in bold.

Following foetal loss, all four mares recovered quickly. Of the 59 close contact mares, 58 mares recorded 58 live healthy foals and one mare died pre‐foaling due to non‐related pathology. There was no statistically significant difference in foal survival between the two groups that did (*n* = 33) and did not (*n* = 26) receive the antimicrobial intervention (P > 0.05). *C. psittaci* was not detected in any of the close contact mares and foals, with 100% result agreement between the isothermal swab testing and qPCR DNA testing (Table 1).

The pooled foetal and placental samples from the four foetal loss cases were all positive for *C. psittaci*, including the 100% result agreement between the isothermal swab testing of foetal C and D samples and qPCR DNA testing (Table 1). In three pooled foetal and placental samples from the *C. psittaci* foetal loss cases we identified *C. psittaci* ST24/*omp*A genotype A strains, sharing 100% sequence identity. These same clonal strains were previously identified in foetal losses from this farm, as well as other Australian equines, psittacine and human hosts (Figure 1).^3^, ^4^, ^6^, ^7^

Case studies are important for understanding sporadic causes of disease. In the case of *C. psittaci* and foetal loss, they also provide an opportunity to develop strategies for handling cases of this zoonotic pathogen that guide future practice and provide guidelines for a safe working environment (Appendix S2). Considering the sequence of foetal losses occurring in separate paddocks, and that *C. psittaci* was not identified in any of the samples from in‐contact mares (or foals), this study once again highlights the sporadic nature of chlamydial foetal loss associated with direct (and/or indirect) transmission presumably from psittacine hosts. Equine *C. psittaci* clonal strains are commonly found in psittacines across Australia,^6^ highlighting the need for an on‐farm protocol (Appendix S2) to reduce the risk of large foal mortality from an outbreak scenario. An equine chlamydiosis protocol needs to consider risk factors including proximity to birds, especially psittacines, the potential for horse‐bird interactions, which may be impacted by feeding strategies, and hygiene. Late gestation during wintertime has been found to be a risk factor for increased *C. psittaci* detection in foetal and newborn foals^1^ and aborted material must be considered a potential source of transmission to other late pregnant mares, as has been shown for humans.^2^ However, we also note that our study found no evidence of transmission between horses.

In the event of any foetal loss case, horse stud workers should proceed with caution (e.g., wearing personal protective equipment, and being aware of staff with compromised immunity) until a diagnostic workup has been completed, as handling of chlamydial infected aborted material may result in zoonotic disease.^2^ *C. psittaci* is a notifiable disease in humans, and in birds and mammals in some Australian states and territories^8^ but any suspected equine cases ideally should involve a full diagnostic workup. Swab sampling of affected foetuses, especially lung tissue and placenta have proven the most reliable for molecular detection.^1^, ^3^, ^4^ However, collection of tissue samples for complimentary histopathology is highly recommended.^9^ Nevertheless, in both swab sampling and tissue collection, the operator must remain aware and be compliant for biosecurity risks associated with this pathogen. Molecular typing of the infecting strain is also important to determine whether the introduction of a new strain has occurred. This is relevant for the Hunter Valley region which has had a dominant clonal strain (ST24) present for many years.^3^, ^6^

In this study, we demonstrated the reliability and utility of *C. psittaci* POC diagnostics. For the samples tested, there was 100% result agreement between the POC and external laboratory *C. psittaci* testing. POC results should be considered as preliminary on‐farm testing and laboratory confirmation is recommended. However, the value in a rapid positive POC result may firstly protect workers from this zoonotic infection. The risk of this infection to people in‐contact with affected horses is greater than the risk to horses, as emphasised by this and other studies^1^, ^2^, ^3^ reporting the lack of transmission between horses. Furthermore, POC results may prevent foal mortality which is important considering the length of gestation, value of the progeny and potential for an outbreak scenario. As assessed in our recent study,^5^ *C. psittaci* POC isothermal testing in this study was estimated at AUD 6 per sample (excluding operator wages and equipment costs) and provided results the same day in contrast to the diagnostic laboratory results which may take up to 5 days.

No benefit was found with the use of tetracycline in the close contact group in this study.

However, no close contact mares tested positive for *C. psittaci* and consequently, antimicrobial intervention and foal outcome were found to be independent of each other. *C. psittaci* is readily susceptible to tetracycline, and tetracycline resistance is currently only reported for the related pig pathogen, *C. suis*. However, studies have also shown that the use of tetracycline as a treatment for chlamydial infections in pigs leads to selection for resistant strains.^10^, ^11^ Further equine infection studies are required to help clarify the benefits of the intervention (if any), and prudence will be required for prophylactic antibiotic use in scenarios where a benefit has not been identified.

The four *C. psittaci* foetal loss events recorded on this farm in one season highlight the continued potential for economic loss to the Australian Thoroughbred industry and the growing need for a protocol to approach equine chlamydiosis (Appendix S2).

## Conflict of interest/Funding information

None of the authors of this paper has a financial or personal relationship with other people or organisations that could inappropriately influence or bias the content of the paper.

## Supporting information


**Appendix S1** Mare and foal *C. psittaci* detection by qPCR and isothermal assay for the four clinical cases of *C. psittaci* foal loss and 59 close contact mares and 33 foals. Associated metadata relating to mare's previous reproductive history, swab sample site, clinical signs, tetracycline administration (pos/neg), dates of all foalings and foal status (live/dead).

**Appendix S2** Protocol for *Chlamydia psittaci* foal loss event. This is a proposed on‐farm protocol which discusses risk factors for *C. psittaci* and provide guidelines for sampling and managing a foal loss event.

## References

[1] Anstey S , Lizárraga D , Nyari S et al. Epidemiology of *Chlamydia psittaci* infections in pregnant thoroughbred mares and foals. The Vet J 2021;273:105683. 34148605 10.1016/j.tvjl.2021.105683

[2] Chan J , Doyle B , Branley J et al. An outbreak of psittacosis at a veterinary school demonstrating a novel source of infection. One Health 2017;3:29–33. 28616500 10.1016/j.onehlt.2017.02.003 PMC5454149

[3] Jenkins C , Jelocnik M , Micallef ML et al. An epizootic of *Chlamydia psittaci* equine reproductive loss associated with suspected spillover from native Australian parrots. Emerg Microbes Infect 2018;7(1):1–13. 29765033 10.1038/s41426-018-0089-y PMC5953950

[4] Akter R , Sansom FM , El‐Hage CM et al. A 25‐year retrospective study of *chlamydia psittaci* in association with equine reproductive loss in Australia. J Med Microbiol 2021;70(2):001284. 33258756 10.1099/jmm.0.001284 PMC8131020

[5] Jelocnik M , Nyari S , Anstey SI et al. Real‐time fluorometric and end‐point colorimetric isothermal assays for detection of equine pathogens *C. psittaci* and equine herpes virus 1: Validation, comparison and application at the point of care. BMC Vet Res 2021;17(1):279. 34412635 10.1186/s12917-021-02986-8 PMC8375077

[6] Anstey SI , Kasimov V , Jenkins C et al. *Chlamydia psittaci* ST24: Clonal strains of one health importance dominate in Australian horse, bird and human infections. Pathogens 2021b;10(8):1015. 34451478 10.3390/pathogens10081015 PMC8401489

[7] Jelocnik M , Branley J , Heller J et al. Multilocus sequence typing identifies an avian‐like *Chlamydia psittaci* strain involved in equine placentitis and associated with subsequent human psittacosis. Emerg Microbes Infect 2017;6(1):1–3. 10.1038/emi.2016.135 PMC5322323 28196971

[8] Australian Institute of Health and Welfare . National Notifiable Diseases Surveillance System. Available online: http://www9.health.gov.au/cda/source/cda-index.cfm (accessed on 10 February 2022).

[9] Macleay CM, Carrick J, Shearer P, et al. A scoping review of the global distribution of causes and syndromes associated with Mid‐ to Late‐term pregnancy loss in horses between 1960 and 2020. Vet Sci 2022;9(4):186. 35448683 10.3390/vetsci9040186 PMC9032147

[10] Cui CY , Chen Q , He Q et al. Transferability of tigecycline resistance: Characterization of the expanding Tet(X) family. WIREs Mech Dis 2022;14(1):e1538. 35023325 10.1002/wsbm.1538

[11] Seth‐Smith HM , Wanninger S , Bachmann N et al. The *Chlamydia suis* genome exhibits high levels of diversity, plasticity, and mobile antibiotic resistance: Comparative genomics of a recent livestock cohort shows influence of treatment regimes. Genome Biol Evol 2017;9(3):750–760. 28338777 10.1093/gbe/evx043 PMC5381551

