# Supplementary material for: Suspected chlamydial foetal loss highlights the need for standardised on‐farm protocols
Source: Aust Vet J. 2022 Sep 7;100(12):600–4. doi: 10.1111/avj.13206 (PMC10087770; doi:10.1111/avj.13206)
Supplement: Supplementary file 2 — Appendix S2 Protocol for Chlamydia psittaci foal loss event. This is a proposed on‐farm protocol which discusses risk factors for C. psittaci and provide guidelines for sampling and managing a foal loss event. [file AVJ-100-600-s002.docx]

**Appendix S2: Proposed protocol for *Chlamydia psittaci* foetal loss event**

**Risk factors for veterinarians and attending staff:** *Chlamydia psittaci* is a zoonotic pathogen, posing a risk to people in-contact with infected horses and birds. Handling of chlamydial infected aborted material or with affected horses may result in a zoonosis and appropriate awareness, education about the pathogen and precaution is advised. *C. psittaci* is a notifiable disease in humans in Australia.

**Risk factors for foetal loss:** Chlamydial foetal loss can occur suddenly on a farm. Risks include proximity to psittacine and extended contact with psittacine, and on farm management practices that may increase horse-bird interactions such as feeding strategies and late gestation during wintertime. Aborting and/or infected mares typically display no clinical sign of ill health.

1. In the event of foetal loss be aware of *C. psittaci* and proceed with caution (alert attending staff and implementing biosecurity measures) until a diagnostic workup has been completed.
2. The staff should wear personal protective equipment (PPE: safety goggles, masks, gloves) and proceed with caution when dealing with and/or collecting samples from affected horses. Masks and gloves should be immediately disposed after the contact with affected horse and/or aborted material, and safety goggles decontaminated with Virkon or 80% ethanol.
3. In case of exposure, humans can remain asymptomatic or may develop usually mild disease, however in some people, particularly the elderly and immunocompromised, it can produce severe illness. Mild to moderate symptoms can develop after 5 – 14 days post exposure, and may include fever, headache, aches, chills and dry cough. Seek medical help if any of symptoms occur.
4. The aborting pregnant mare may not show any clinical signs; treat as per on farm veterinary protocol.
5. Samples: Obtain dry swabs (Copan Sterile Minitip Rayon Swab w/ Aluminium Applicator) of the tissues of affected foetus (e.g., lung) and placenta. These sites have proven the most reliable for chlamydial qPCR detection. Other swabs, such as those with Universal Transport Medium, can be also used. Upon collection, store swabs at 4 ^o^C if more immediate testing if available (24 – 48h), or at -20 ^o^C if there are delays (up to a week) for testing. Samples kept unfrozen beyond this timepoint may have reduced detection sensitivity.
6. Where possible (e.g., if a stud has onsite veterinary team and a laboratory space), consider implementing rapid point of care *C. psittaci* diagnostics, such as the Chlamydia LAMP assay, to provide faster diagnosis in suspected chlamydia foetal loss and to alert the attending staff. Test swabs as soon as possible.
7. Standard laboratory qPCR testing of swab samples is also recommended for definitive diagnosis of chlamydial foetal loss.
8. Mare nasal and vaginal swabs for chlamydial qPCR detection and/or blood for serology have proved unreliable for diagnostics to date. These may not to be collected.
9. Histopathology is an additional procedure for diagnostic confirmation. Sampling of foetal and placental tissues at postmortem, stored in 10% buffered formalin.
10. Move close contact mares into a new paddock away from the aborting mare and potential contact with infected foetal membranes. However, do note that these practices remain anecdotal. At present, there is no evidence of chlamydial transmission between horses.
11. Consider appropriate antimicrobial stewardship. At present, there is no evidence for prophylactic antibiotic use in close-contact mares.
12. Molecular typing of *C. psittaci* positive samples should be considered to identify the infecting strain and assess any outbreak potential with introduction of a new strain. This is done with DNA extracted from swab samples, ideally frozen post collection to preserve DNA.
13. Considered requesting a test for *Chlamydia* if newborn foals, weanlings and yearlings with respiratory illness.
14. During pregnancy, implement preventative measures that minimise horse/bird contacts and maintain clean environment: regularly inspection of feeding and water troughs for ill birds and bird faeces, and cleaning of the feeding and water troughs (e.g., Virkon) in paddocks, and assess feeding regimen to late pregnant mares with relation to bird contact (feed preparation inside the sheds rather in the open and transport the feed covered. However, do note that these practices remain anecdotal.
